# PRMT7: a survive-or-die switch in cancer stem cells

**DOI:** 10.1186/s12943-022-01602-z

**Published:** 2022-06-10

**Authors:** Christophe Nicot

**Affiliations:** Department of Pathology, University of Kansas Medical Center, 3901 Rainbow Boulevard, Kansas City, KS 66160 USA

Cancer stem-like cells (CSCs) are involved in initiation, resistance and relapse of cancer [1, 2]. Identifying targets uniquely expressed in CSCs, instead their normal counterparts, has been puzzling the field of cancer biology. In the quintessential paradigm of CSCs in chronic myeloid leukemia (CML), resistance at least partially conferred by leukemia stem cells (LSCs) remains an increasing threat in patients diagnosed with CML [3, 4] despite tyrosine kinase inhibitors such as Imatinib mesylate magically prolonging the survival and improving life quality. In a recent issue of *Cell Metabolism*, Liu et al. [5] have demonstrated that protein arginine methyltransferase 7 (PRMT7) get hold of a survive-or-die switch in CML LSCs. Indeed, targeting PRMT7 by either genetic or pharmacological approaches selectively eliminate LSCs while sparing normal counterpart cells. The mechanism has been well illustrated. These findings have brought us a phenomenal therapeutic target for CSCs.

Epigenetics provide rooms for recognizing the intricate regulation of stemness [6–8]. In their studies, Liu et al. [5] chose PRMT7 based on differential expression in CML LSCs, and authors took advantages of genetic and pharmacological tactics to comprehensively characterize the function of PRMT7 in survival and self-renewal of CML LSCs. First, they generated hematopoietic cell-specific *Prmt7* knockout mice to investigate the role of *Prmt7* in leukemogenesis of BCR-ABL-driven CML. Interestingly, the authors found that conditional knockout (CKO) of *Prmt7* markedly delayed the development of leukemia, and significantly mitigated leukemia burden in CML mice. Of note, *Prmt7* CKO dramatically reduced the populations of leukemia stem/progenitor cells (LSPCs) and the frequency of LSCs. *Prmt7* CKO also suppressed the CFC/replating capacity of LSCs and the disease reconstitution ability of LSCs in secondary recipients. These lines of compelling evidence indicate that PRMT7 is required for the self-renewal of LSCs. In the meantime, *Prmt7* CKO did not impact either the frequency of hematopoietic stem cells (HSCs), or their function of normal HSCs.

After obtaining evidence that PRMT7 regulates LSCs in a methyltransferase catalysis-dependent manner, the authors set out to develop small molecule inhibitors against PRMT7. Authors successfully synthesized a small inhibitor, JS1310, which selectivity inhibited PRMT7 but not other PRMT family members. In vivo studies using JS1310 treatment prominently eliminated LSCs and prolonged survival of CML mice. Most importantly, authors also investigated the effects of JS1310 on primary CD34^+^ cells from CML patients or healthy donors. JS1310 profoundly suppressed the survival and self-renewal ability of CML CD34^+^ cells without affecting normal CD34^+^ cells. In a patient-derived xenograft model, JS1310 treatment showed a significant inhibition in the long-term engraftment capacity of human CML CD34^+^ cells. Overall, PRMT7 may represent a unique and valuable therapeutic target against CML LSCs.

To gain insight into the underlying mechanism, the authors did RNA-seq analysis and found that glycine decarboxylase (GLDC) [9, 10], a rate-limiting enzyme in glycine metabolism regulation network, was significantly downregulated in *Prmt7*-CKO LSCs compared to wild-type counterparts. Rescue experiments revealed that GLDC regulated LSCs in an enzyme activity-dependent manner. Furthermore, ChIP assay showed that the symmetric methylation of H2AR3 by PRMT7 decreased the enrichment of TRPS1 at the promoter region of GLDC, suggesting that transcriptional repressor GATA binding 1 (TRPS1) may be a mediator for PRMT7 to regulate GLDC. Surprisingly, PRMT7 CKO did not alter TRPS1 and GLDC expression in normal HSCs. This may provide a well and helpful explanation for the phenotype that loss of PRMT7 is not detrimental to normal HSCs.

Gene Ontology analysis revealed that genes related glycine and serine metabolism pathway were significantly enriched in the *Prmt7* CKO LSCs. High pressure-liquid chromatography-mass spectrometry analysis indicated a progressive increase in intracellular glycine and serine with progression of CML disease in CML mice. Besides, loss of PRMT7 resulted in excessive accumulation of glycine in LSCs.

Glycine generated from serine by serine hydroxymethyltransferase 2 (SHMT2) can be converted to either non-toxic 5, 10-methylene-tetrahydrofolate (5, 10-MTHF) by GLDC or toxic methylglyoxal by glycine C-acetyltransferase (GCAT) [11, 12]. The authors hypothesized that the increased apoptosis in LSCs might be implemented by toxic methylglyoxal which is converted from excess glycine in *Prmt7*-CKO LSCs. As expected, methylglyoxal level was remarkably increased in *Prmt7*-CKO LSCs from CML mice or PRMT7 inhibitor-treated CD34^+^ cells from patients with CML. Methylglyoxal treatment indeed suppressed survival and self-renewal of LSCs. *Prmt7*-CKO CML mice fed with serine and glycine-free (SG-free) diet exhibited shorted survival than those fed with control diet. SG-free diet also reversed the *Prmt7* CKO-mediated decrease in leukemia cells and LSPCs.

Taken together, the authors proposed the working model (Fig. 1). In the context of intact PRMT7 overexpressed in LSCs, glycine metabolism affluently provides 5, 10-MTHF allowing LSCs metabolic addiction. In the context of PRMT7 deletion or inhibition, however, not only the pro-survival of metabolic addiction was blocked but also the glycine metabolism is reshaped to generate excessive methylglyoxal, which is poisonous to LSCs. In the case of normal HSCs absent of PRMT7 overexpression, inhibition of PRMT7 would not kill normal HSCs because of lack of the target. These findings have underscored that PRMT7 is a promisingly selective target of LSCs, and greatly improved our understanding on epigenetic regulation of CSCs. However, relevant question like how PRMT7 is overexpressed in CML LSCs remain to be addressed in future.

**Fig. 1:**
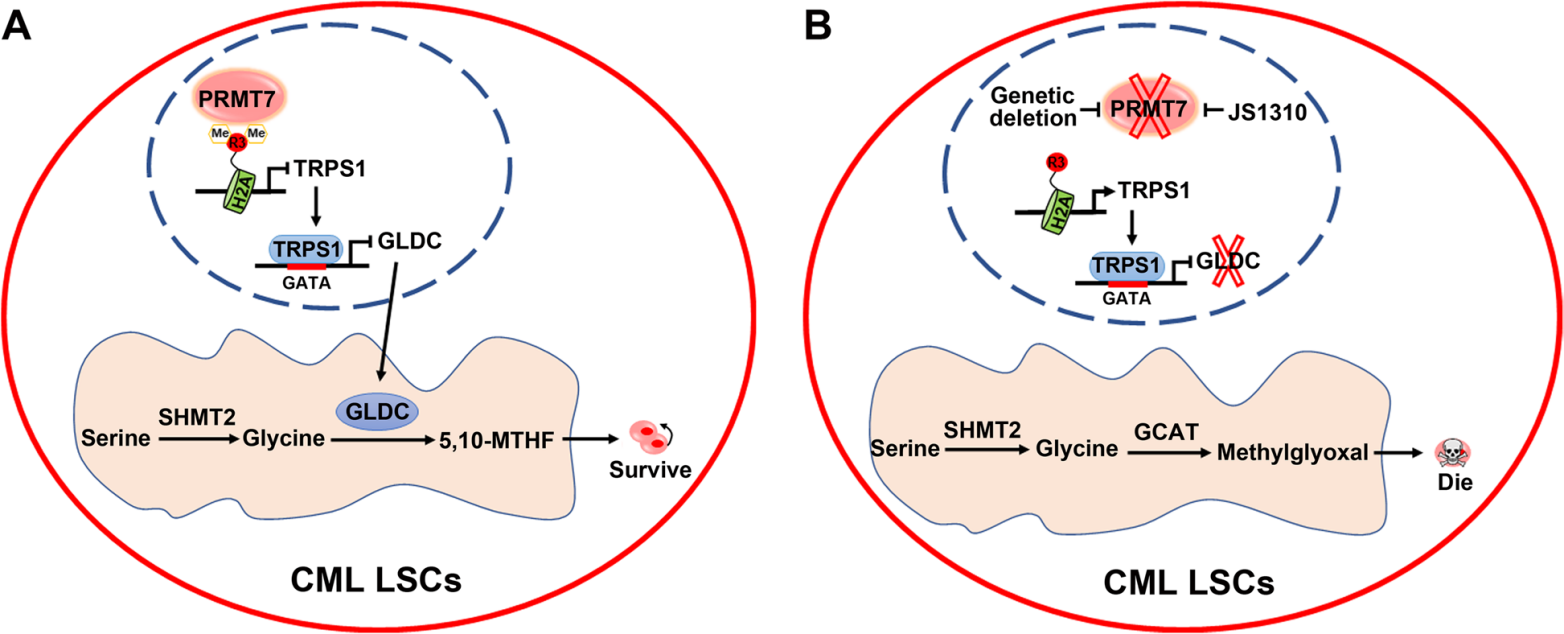
PRMT7 seizes glycine metabolism switch to determine fate of LSCs. **A** PRMT7 catalyzes H2AR3me2s to inhibit the transcription repressor of TRPS1, eventually upregulating GLDC in CML LSCs. Glycine generated from serine by SHMT2 is converted by GLDC to 5, 10-MTHF, allowing LSCs to gain metabolic addiction. **B** Genetic deletion or pharmacological inhibition of PRMT7 derepresses the repressor TRPS1, thereby downregulates GLDC and subsequently accumulates intracellular glycine in CML LSCs. Increased intracellular glycine is converted by GCAT to methylglyoxal, which induces the death of CML LSCs

## References

[1] Prager BC, Xie Q, Bao S, Rich JN (2019). Cancer stem cells: the architects of the tumor ecosystem. Cell Stem Cell.

[2] Batlle E, Clevers H (2017). Cancer stem cells revisited. Nat Med.

[3] Braun TP, Eide CA, Druker BJ (2020). Response and resistance to BCR-ABL1-targeted therapies. Cancer Cell.

[4] Vetrie D, Helgason GV, Copland M (2020). The leukaemia stem cell: similarities, differences and clinical prospects in CML and AML. Nat Rev Cancer.

[5] Liu C, Zou W, Nie D, Li S, Duan C, Zhou M, et al. Loss of PRMT7 reprograms glycine metabolism to selectively eradicate leukemia stem cells in CML. Cell Metab. 2022;S1550-4131(22):00131-0. 10.1016/j.cmet.2022.04.004. Online ahead of print. 10.1016/j.cmet.2022.04.004 35508169

[6] Zhou J, Nie D, Li J, Du X, Lu Y, Li Y, Liu C, Dai W, Wang Y, Jin Y, Pan J (2018). PTEN is fundamental for elimination of leukemia stem cells mediated by GSK126 targeting EZH2 in chronic myelogenous leukemia. Clin Cancer Res.

[7] Jin Y, Zhou J, Xu F, Jin B, Cui L, Wang Y, Du X, Li J, Li P, Ren R, Pan J (2016). Targeting methyltransferase PRMT5 eliminates leukemia stem cells in chronic myelogenous leukemia. J Clin Invest.

[8] Zhou J, Wang S, Nie D, Lai P, Li Y, Li Y, Jin Y, Pan J (2021). Super-enhancer landscape reveals leukemia stem cell reliance on X-box binding protein 1 as a therapeutic vulnerability. Sci Transl Med.

[9] Zhang WC, Shyh-Chang N, Yang H, Rai A, Umashankar S, Ma S, Soh BS, Sun LL, Tai BC, Nga ME (2012). Glycine decarboxylase activity drives non-small cell lung cancer tumor-initiating cells and tumorigenesis. Cell..

[10] Alptekin A, Ye B, Yu Y, Poole CJ, van Riggelen J, Zha Y, Ding HF (2019). Glycine decarboxylase is a transcriptional target of MYCN required for neuroblastoma cell proliferation and tumorigenicity. Oncogene..

[11] Kim D, Fiske BP, Birsoy K, Freinkman E, Kami K, Possemato RL, Chudnovsky Y, Pacold ME, Chen WW, Cantor JR (2015). SHMT2 drives glioma cell survival in ischaemia but imposes a dependence on glycine clearance. Nature..

[12] Locasale JW (2013). Serine, glycine and one-carbon units: cancer metabolism in full circle. Nat Rev Cancer.

